# Prevalence of malocclusion in public school students in the mixed dentition phase and its association with early loss of deciduous teeth

**DOI:** 10.1590/2177-6709.27.4.e2220120.oar

**Published:** 2022-09-23

**Authors:** Marianella Aguilar Ventura FADEL, Bianca Zimmermann SANTOS, Raquel Pippi ANTONIAZZI, Leonardo KOERICH, Vera Lúcia BOSCO, Arno LOCKS

**Affiliations:** 1 Universidade Federal de Santa Catarina, Centro de Ciências Biológicas, Departamento de Odontologia, (Florianópolis/SC, Brazil).; 2 Universidade Franciscana, Departamento de Odontologia (Santa Maria/RS, Brazil).; 3 Universidade Federal de Santa Maria, Departamento de Estomatologia, Faculdade de Odontologia (Santa Maria / RS, Brazil).; 4 University of North Carolina, Department of Dentistry (Charlotte/NC, USA).

**Keywords:** Malocclusion, Mixed dentition, Public health

## Abstract

**Objective:**

To determine the prevalence of malocclusion and its association with the early loss of deciduous teeth and other factors in children in the mixed dentition phase, aged six to eight years, enrolled in public schools in southern Brazil.

**Methods:**

A cross-sectional study was conducted with a representative sample of 528 children from municipal public schools in 2009. Data collection involved a clinical examination for the determination of early tooth loss, dental caries, tongue pressure and malocclusion (outcome), as well as the administration of a questionnaire. Poisson regression analysis with robust variance was used to model the association between malocclusion and the independent variables.

**Results:**

The prevalence of malocclusion and early tooth loss was 69.1% and 21.8%, respectively. In the raw data analysis, malocclusion was associated with age, early tooth loss, dental caries and tongue pressure. After the adjustment, the likelihood of malocclusion was greater among children older than eight years, those who exerted tongue pressure on the teeth and those with early tooth loss. The likelihood of malocclusion was 24% greater among children with early tooth loss, compared to those without tooth loss.

**Conclusion:**

The early loss of deciduous teeth was associated with the occurrence of malocclusion in the children studied.

## RESUMO

**Objetivo:**

Determinar a prevalência de má oclusão em crianças de seis a oito anos de idade no município de Florianópolis (Santa Catarina, Brasil), e a sua associação com a perda precoce de dentes decíduos.

**Métodos:**

Esse estudo transversal avaliou uma amostra representativa de 528 crianças de escolas públicas municipais de Florianópolis, no ano de 2009. Os dados foram coletados por meio de exame clínico (perda dentária precoce, cárie dentária, interposição lingual e má oclusão - que foi o desfecho do estudo) e de um questionário. A regressão de Poisson com variância robusta foi utilizada para modelar a associação entre má oclusão e as variáveis independentes.

**Resultados:**

A prevalência de má oclusão e de perda dentária precoce foi, respectivamente, de 66,7% e 21,8%. Na análise dos dados brutos, a má oclusão foi associada com a idade, perda dentária precoce e cárie dentária. Após ser feito o ajuste, crianças com mais de 7 anos, com interposição lingual e perda dentária precoce apresentaram maior probabilidade de ter má oclusão. Crianças com perda dentária precoce apresentaram probabilidade 24% maior de ter má oclusão do que aquelas sem perda dentária.

**Conclusões:**

A perda precoce de dentes decíduos foi associada à ocorrência de má oclusão nas crianças estudadas.

## INTRODUCTION

Regardless of the developmental stage of the dentition, researchers are unanimous in declaring that the prevalence of malocclusion is higher than normal occlusion, in different populations.^1^ ^-^ ^4^ Due to the high prevalence and impact on quality of life, malocclusions are considered a global public health problem.^5^ ^,^ ^6^

Epidemiological studies demonstrated that the frequent occurrence of malocclusion is related to its multifactorial etiology, with the combined influence of genetic and environmental factors.^2^ ^,^ ^3^ ^,^ ^7^ ^,^ ^8^ The early loss of deciduous teeth, especially molars, is one of the etiological factors of malocclusions.^9^ ^,^ ^10^ Although traumatic dental injury can lead to tooth loss, dental caries continue to be the major cause.^11^ ^-^ ^14^

The transition from the deciduous to the permanent dentition is a complex phenomenon in which deciduous teeth play an important role. The early loss of these teeth can cause the migration of adjacent teeth, leading to a reduction or even the complete closure of the space destined for the eruption of the successor permanent teeth, and affecting the chronology of the eruption process.^9^ ^,^ ^15^ Although the understanding of the mixed dentition and the repercussion of early tooth loss is important to the planning of preventive and interceptive measures, few studies in the literature have evaluated this phase. The diagnosis and early treatment of occlusal abnormalities in any phase of the dentition favor adequate growth and development, reduce the cost, time and severity of orthodontic treatment, and favor planning in the public health area.^16^

The following hypotheses were tested in the present study: A) the prevalence of malocclusion is high in the mixed dentition phase; and B) there is an association between malocclusion and the early loss of deciduous teeth in the mixed dentition phase. Therefore, the aim of this study was to determine the prevalence of malocclusion and the association with the early loss of deciduous teeth and other factors in children in the mixed dentition phase, aged six to eight years, enrolled in public schools in the municipality of Florianópolis, Santa Catarina, Brazil.

## METHODS

An observational cross-sectional study was conducted between March and July 2009, with a representative sample of children in the mixed dentition phase, aged six to eight years, enrolled in public schools in the municipality of Florianópolis, Santa Catarina, Brazil. 

The estimated population of the city in the period was approximately 408 thousand residents, with 23,974 children aged five to nine years. The sample size was determined using a sampling procedure considering a total of 7,636 students from 32 public schools distributed among the five regions of interest (northern, southern, eastern, western and central). Cluster sampling was performed. The primary sampling unit was public schools in the municipality, and 24 of the 32 schools distributed in the five administrative regions of Florianópolis were randomly selected (by lots), considering the proportion of the population that attended each school. In the northern, southern and eastern regions, one class was randomly selected from each school to ensure that students from all grades would be selected in each region. In the western and central regions, all students in all grades were selected. This procedure resulted in a sample of 528 children in the target age range enrolled in 24 municipal public schools. 

The inclusion criteria were: enrollment in a primary school in the municipal public school system in the first semester of 2009, age six to eight years, being in the mixed dentition phase and not wearing an orthodontic appliance. 

Data collection involved the use of a structured questionnaire administered to parents/caregivers in interview format, addressing general information and dental characteristics of the children, as well as a clinical examination of the children, performed by an examiner who had undergone a training and calibration exercise. Training and calibration were performed for malocclusion, and consisted of theoretical evaluation of clinical images, a discussion of each category and possible disagreements. Training was concluded when a good level of agreement and understanding was achieved. Intra-examiner reproducibility was determined through the assessment of 25 children with duplicate exams, separated by a one-week interval (Kappa = 0.93). Inter-examiner reproducibility between the examiner and the gold standard examiner was determined by examining the same patients (Kappa = 0.89).

Data on nail biting and thumb/finger sucking were collected by means of a questionnaire administered to the parents/caregivers, and the habit of lip/tongue interposition was determined during the clinical examination, which was performed under natural and artificial light, with the aid of disposable tongue depressors and relative isolation.^18^ Malocclusion was recorded in the presence of an Angle Class II or Class III molar relationship or a Class I molar relationship associated with at least one of the following: anterior open bite, anterior and/or posterior crossbite, accentuated overjet and/or overbite (> 3 mm) and dental crowding.^19^ Tooth loss was classified as premature when a deciduous tooth was extracted at least one year prior to the eruption of the successor permanent tooth.^19^ This information was obtained from the parents/caregivers. Caries experience was evaluated using the mean value of the Decayed, Missing and Filled Teeth (DMFT) index. Flat dental mirrors, a community periodontal index (CPI) probe and natural and artificial light^17^ were used to evaluate all dental surfaces. Tongue interposition was investigated by the observation of water swallowing (without touching the participant) and checking for tongue projection and the participation of the perioral musculature, such as contraction of the lips and the mental muscle.^18^ Lip interposition was assessed by observing the child.^18^

The data were analyzed using SPSS for Windows version 15.0. Descriptive statistics involved calculation of the distribution of relative frequencies, to identify the percentage of prematurely lost deciduous teeth and malocclusion categories, and obtain prevalence rates for the other variables of interest. The chi-square test was used to determine associations between the variables investigated and child’s sex. Raw and adjusted Poisson analyses were performed, with the determination of prevalence ratios (PR) and respective 95% confidence intervals (CI). In the adjusted model, malocclusion was the dependent variable, and the independent variables were sex (female or male), age (six, seven or eight years), early tooth loss (no/yes), dental caries (no/yes), nail biting habit (no/yes), thumb sucking (no/yes), lip pressure (no/yes) and tongue pressure (no/yes). The forward selection process was used for the determination of variables to be incorporated into the model, for which the inclusion criterion was a p-value < 0.25 in the raw analysis (Wald test). The significance level was set to 5% for all statistical tests. 

This study received approval from the local Human Research Ethics Committee at the *Universidade Federal de Santa Catarina* (certificate number: 029/2009).

## RESULTS

The response rate was 71% (528/743). Among the 528 children evaluated, 69.1% (n = 365) exhibited malocclusion and 21.8% (n = 115) had early deciduous tooth loss. And 25.8% and 3.0% of the participants had Angle Classes II and III, respectively. Dental caries were found in 33.5% (n = 177). Among the deleterious oral habits investigated, the highest frequencies were found for nail biting (54.4%; n = 287) and tongue pressure (38.3%; n = 202) (Table 1). 

**Table 1:** Prevalence of age groups, malocclusion, early tooth loss, dental caries and deleterious oral habits among children analyzed, stratified by sex (Florianópolis, Brazil, 2009).

Variables	General		Boys		Girls		p*
	n	%	n	%	n	%	
Age (years)							
6	150	28.4	83	55.3	67	44.7	0.316
7	216	40.9	103	47.7	113	52.3	
8	162	30.7	86	53.1	76	46.9	
Malocclusion							
No	182	33.3	82	46.3	95	53.7	0. 055
Yes	365	66.7	190	54.1	161	54.1	
Early tooth loss							
No	413	78.2	207	80.9	206	75.7	0. 154
Yes	115	21.8	49	19.1	66	24.3	
Dental caries							
No	351	66.5	166	64.8	185	68	0. 440
Yes	177	33.5	90	35.2	87	32	
Nail biting							
No	241	45.6	113	44.1	128	45.6	0. 501
Yes	287	54.4	143	55.9	144	54.4	
Thumb sucking							
No	456	86.4	226	88.3	230	84.6	0. 213
Yes	72	13.6	30	11.7	42	15.4	
Lip pressure							
No	516	97.7	252	98.4	264	97.1	0. 288
Yes	12	2.3	4	1.6	8	2.9	
Tongue pressure							
No	326	61.7	169	66	157	57.7	0. 050
Yes	202	38.3	87	34	115	42.3	
Total	528	100	272	51.5	256	48.5	

* chi-square test.

Early tooth loss was not significantly associated with the types of Angle malocclusion (*p*= 0.139). Table 2 shows that girls (73.2%), eight-year-old children (87.7%), with deciduous tooth loss (97.4%), with dental caries (75.1%) and with excessive tongue pressure (77.7%) had the highest prevalence of malocclusion. The prevalence of malocclusion was slightly lower among participants with early tooth loss (97.4%), dental caries (75.1%), nail biting (70.0%), thumb sucking (56.9%) and lip pressure (66.7%).

**Table 2:** Demographic, behavioral and clinical characteristics, according to malocclusion. (Florianópolis, Brazil, 2009).

Variables	Malocclusion			
	No		Yes	
	n	%	n	%
Sex				
Male	90	35.2	166	64.8
Female	73	27.8	199	73.2
Age				
6 years	68	45.3	82	54.7
7 years	75	34.7	141	65.3
8 years	20	12.3	142	87.7
Early tooth loss				
No	160	38.7	253	61.3
Yes	3	2.6	112	97.4
Dental caries				
No	119	33.9	232	66.1
Yes	44	24.9	133	75.1
Nail biting				
No	77	32.0	164	68.0
Yes	86	30.0	201	70.0
Thumb sucking				
No	132	35.7	324	64.3
Yes	31	43.1	41	56.9
Lip pressure				
No	159	30.8	357	69.2
Yes	4	33.3	8	66.7
Tongue pressure				
No	118	36.2	208	63.8
Yes	45	22.3	157	77.7
Total	163	30.9	365	69.1

The prevalence of malocclusion was higher among the eight-year-old children who had deciduous tooth loss and exerted tongue pressure (*p*< 0.05). In the analysis with malocclusion as the dependent variable, age (PR = 1.11; 95% CI: 1.04 to 1.72), early tooth loss (PR = 1.24; 95% CI: 1.19 to 1.28) and tongue pressure (PR = 1.09; 95% CI: 1.04 to 1.13) remained associated with the outcome in the adjusted analysis (Table 3). 

**Table 3:** Raw and adjusted prevalence ratios (PR) for malocclusion, according to independent variables (Florianópolis, Brazil, 2009).

Variables	Malocclusion					
	Raw analysis*			Adjusted analysis*		
	PR	95% CI	p**	PR	95% CI	p**
Sex			0.139			
Male	1.00					
Female	1.13	0.99-1.88				
Age			< 0.001			< 0.001
6 years	1.00			1.00		
7 years	1.06	1.01-1.14		1.07	1.01-1.14	
8 years	1.15	1.09-1.23		1.11	1.04-1.72	
Early tooth loss			< 0.001			< 0.001
No	1.00			1.00		
Yes	1.41	1.21-1.49		1.24	1.19-1.28	
Dental caries			0.006			
No	1.00					
Yes	1.09	1.02-1.12				
Nail biting			0.882			
No	1.00					
Yes	1.01	0.96-1.06				
Thumb sucking			0.719			
No	1.00					
Yes	0.99	0.92-1.06				
Lip pressure			0.996			
No	1.00					
Yes	0.99	0.85-1.18				
Tongue pressure			0.002			< 0.001
No	1.00			1.00		
Yes	1.19	1.03-1.30		1.09	1.04-1.13	

* Poisson regression with robust variance; **Wald test, statistically significant difference at p < 0.05.

## DISCUSSION

In the present study, the prevalence of malocclusion in the mixed dentition was high and was associated with the early loss of deciduous teeth. These findings are in agreement with data described in previous studies reporting a high frequency of malocclusion in this phase.^16^ ^,^ ^21^ Moreover, the early loss of deciduous teeth causes harm that can extend to the permanent dentition and affect the development of bone and muscle tissues of the face, thereby contributing to the establishment and/or aggravation of malocclusion, as well as causing problems related to speaking, chewing, aesthetics, psychological aspects and the appearance of deleterious oral habits.^9^ ^,^ ^10^ ^,^ ^22^ ^,^ ^23^ These findings can be used to guide decision making by the dentists, and can contribute to public health measures, especially those targeting more vulnerable populations, with a low socioeconomic status, as in the sample of the present study.

An association was found between the ages of seven and eight years and a greater prevalence of malocclusion. According to previous studies, early tooth loss at younger ages leads to a greater reduction in the perimeter of the dental arch.^24^ In the present study, there was no record of when tooth loss occurred, and the seven-year-old and eight-year-old children may have prematurely lost their deciduous teeth years earlier. They may also have accumulated early losses and other alterations in facial and dental development that could explain the association encountered. 

Dental caries were found in less than half of the children, and no longer presented a significant association with malocclusion in the adjusted analysis. A cross-sectional study conducted with 646 Chinese children aged 6 to 13 years found an association between caries and malocclusion.^25^ Another study involving 1,601 children aged 12 to 14 years found a 63.8% prevalence rate of malocclusion, and the association with caries experience was confirmed in the multivariate analysis.^26^ It is important to point out that the DMFT index used in the present study to measure dental caries does not distinguish between a small, slightly cavitated lesion and another with considerable loss of dental structure, which may have influenced the results, as caries only has a significant effect on the occlusion when it causes the loss of tooth structure, especially in the interproximal region.^25^

Among the deleterious oral habits investigated, only tongue pressure was associated with malocclusion. The swallowing pattern is commonly altered in children who use a pacifier or are bottle fed with greater duration and frequency or who have early tooth loss in the anterior region.^11^ Moreover, the occurrence of tongue pressure is mainly associated with anterior open bite.^27^ Some etiological factors, including early tooth loss, can lead to an improper swallowing pattern, altering tongue posture. This pattern may imply lingual pressure on the anterior and posterior teeth, causing changes such as anterior open bite, increased overjet and posterior crossbite.^28^

It is also necessary to consider that the consequences of early deciduous tooth loss are influenced by many factors, such as the age at which the loss occurred, potential facial and dental growth, and the occurrence of deleterious oral habits.^11^ Tooth movement depends on the eruption space, time and route, intercuspation and age at the time of tooth extraction.^24^ Thus, the management of the space after early tooth loss plays an important role in preventive and interceptive orthodontics.^24^ The use of space maintainers in the developing dentition can help prevent the loss of length and width in the dental arch, especially in cases of early loss of the deciduous molars. 

Some limitations of this study should be considered when interpreting the results. The assessment of the early loss of deciduous teeth by means of parents’ reports increases the risk of memory bias. It is also important to consider that some factors that were not evaluated can influence the severity of the consequences of early loss of deciduous teeth. The Dental Aesthetic Index (DAI) is not sensitive to some occlusal problems detected using the Angle classification and *vice versa*, demonstrating that both indices have distinct strengths in the detection of malocclusion and should be used in a complementary manner.^29^ Regarding dental caries, no interproximal radiographs were taken for the diagnosis of hidden caries. However, according to the Oral Health Survey Basic Methods,^17^ radiographs are not necessary, and dental caries should be measured by means of a clinical examination of the dentition and treatment needs of the deciduous and permanent teeth. Additionally, some variables that could exert an influence on the outcome were not collected, such as socioeconomic factors, pacifier use and bottle use - the latter two of which could have influenced the establishment of malocclusion. 

Despite these limitations, the present results have clinical and scientific importance. The findings can be used to guide decision making on the part of dentists, and can contribute to public health measures. The creation of the *Programa Brasil Sorridente* (Brazil Smiling Program) and the inclusion of oral health in the *Programa Saúde da Família (*Family Health Program) were important to the broadening of access to dental services for the Brazilian population through the public healthcare system. However, Orthodontics and Pediatric Dentistry are specialties in Brazil that have historically been provided by the private sector. Thus, considering the high prevalence of malocclusion in childhood^30^ and the negative repercussions of the early loss of deciduous teeth, we suggest the inclusion of health professionals from these fields working together in the public health area. The offer of integral oral health services for children at public health services will enable joint actions between Orthodontics and Pediatric Dentistry. These actions, as well as the early diagnosis and treatment of oral problems such as caries, can prevent early tooth loss or enable treatment through preventive Orthodontics, resulting in the prevention of malocclusions, which require more complex, expensive, time-consuming treatments. Actions of this type can exert a positive impact on oral health-related quality of life in children and their families.^31^

## CONCLUSION

More than half of the evaluated children exhibited malocclusion, which was associated with the early loss of deciduous teeth, even after the adjustment for the independent variables.
